# Mechanistic considerations for the use of monoclonal antibodies for cancer therapy

**DOI:** 10.7497/j.issn.2095-3941.2014.01.002

**Published:** 2014-03

**Authors:** Patrick M. Glassman, Joseph P. Balthasar

**Affiliations:** Department of Pharmaceutical Sciences, School of Pharmacy and Pharmaceutical Sciences, University at Buffalo, The State University of New York, Buffalo, NY 14214, USA

**Keywords:** Antibodies, monoclonal, oncology, pharmacokinetics, pharmacodynamics

## Abstract

Since the approval of rituximab in 1997, monoclonal antibodies (mAbs) have become an increasingly important component of therapeutic regimens in oncology. The success of mAbs as a therapeutic class is a result of great strides that have been made in molecular biology and in biotechnology over the past several decades. Currently, there are 14 approved mAb products for oncology indications, and there are ten additional mAbs in late stages of clinical trials. Compared to traditional chemotherapeutic agents, mAbs have several advantages, including a long circulating half-life and high target specificity. Antibodies can serve as cytotoxic agents when administered alone, exerting a pharmacologic effect through several mechanisms involving the antigen binding (Fab) and/or Fc domains of the molecule, and mAbs may also be utilized as drug carriers, targeting a toxic payload to cancer cells. The extremely high affinity of mAbs for their targets, which is desirable with respect to pharmacodynamics (i.e., contributing to the high therapeutic selectivity of mAb), often leads to complex, non-linear, target-mediated pharmacokinetics. In this report, we summarize the pharmacokinetic and pharmacodynamics of mAbs that have been approved and of mAbs that are near approval for oncology indications, with particular focus on the molecular and cellular mechanisms responsible for their disposition and efficacy.

## Introduction

The use of antibodies in oncology dates back to the work of Héricourt and Richet, who in the late 1800s, described serotherapy as a potential approach to treating malignancies^1^. Several decades later, Paul Ehrlich proposed the ‘magic bullet’ hypothesis, which suggested that drugs could be developed that were highly selective for pathogenic cells, thereby granting the drug high potency with minimal off-site toxicity^2^. Despite this early work, it was not until Köhler and Milstein described hybridoma technology in 1975^3^ that development of monoclonal antibodies (mAbs) became a feasible approach in drug development. In the decades immediately after this breakthrough, coinciding with the advent of improved molecular biology techniques, it became possible for mAbs to be generated with increasing proportions of ‘human’ content. As a result, investigators are now able to produce chimeric, humanized, and fully human mAbs. Antibody platforms which incorporate human regions provide many benefits over the initially developed rodent mAbs, including the potential for reduced immunogenicity, improved effector function, and improved pharmacokinetic properties due to higher affinity interactions with the neonatal Fc receptor (FcRn) in humans.

Currently, there are 14 therapeutic mAbs approved by the FDA for use in oncology (**Table 1**). Of these, ten are administered as ‘naked’ mAbs, two are radioimmunoconjugates (ibritumomab tiuxetan and ^131^I tositumomab), and two are antibody-drug conjugates (ADC) (brentuximab vedotin and trastuzumab emtansine). Additionally, as of July 2013, there are 10 mAbs in late-stage (Phase II/III and Phase III) clinical trials^15^ (**Table 2**). These products are utilized as major components in the therapeutic regimens for a wide variety of solid and liquid cancers.

**Table 1:** FDA-approved mAbs for use in oncology

Name	Marketed by	Class	Target	First approved indication	Reported mechanisms of action	Approval year
Rituximab (Rituxan)	Biogen Idec/Genentech	Chimeric IgG1	CD20	Non-Hodgkin’s Lymphoma	ADCC, CDC, Induction of Apoptosis^4^	1997
Trastuzumab (Herceptin)	Genentech	Humanized IgG1	HER2	Breast Cancer	Signal Inhibition, ADCC^5^	1998
Alemtuzumab (Campath)	Sanofi-Aventis	Humanized IgG1	CD52	B cell Chronic Lymphocytic Leukemia	CDC, Induction of Apoptosis^6^	2001
Ibritumomab tiuxetan (Zevalin)	Biogen Idec	Murine IgG1	CD20	Non-Hodgkin’s Lymphoma	Radioisotope Delivery (^90^Y)	2002
Tositumomab (Bexxar)	GlaxoSmithKline	Murine IgG2a	CD20	Non-Hodgkin’s Lymphoma	Radioisotope Delivery (^131^I), ADCC, CDC, Induction of Apoptosis^7^	2003
Cetuximab (Erbitux)	Bristol-Myers Squibb/Eli Lilly	Chimeric IgG1	EGFR	Squamous Cell Carcinoma of the Head and Neck	Signal Inhibition, ADCC, CDC^8^	2004
Bevacizumab (Avastin)	Genentech	Humanized IgG1	VEGF	Colorectal Cancer	Signal Inhibition^9^	2004
Panitumumab (Vectibix)	Amgen	Human IgG2	EGFR	Colorectal Cancer	Signal Inhibition, ADCC^10^	2006
Ofatumumab (Arzerra)	Genmab/GSK	Human IgG1	CD20	Chronic Lymphocytic Leukemia	ADCC, CDC^11^	2009
Denosumab (Xgeva)	Amgen	Human IgG2	RANKL	Bone Metastases	Signal Inhibition	2010
Ipilimumab (Yervoy)	Bristol-Myers Squibb	Human IgG1	CTLA-4	Metastatic Melanoma	Signal Inhibition^12^	2011
Brentuximab vedotin (Adcetris)	Seattle Genetics	Chimeric IgG1	CD30	Hodgkin Lymphoma	ADC	2011
Pertuzumab (Perjeta)	Genentech	Humanized IgG1	HER2	Breast Cancer	Signal Inhibition, ADCC^13^	2012
Trastuzumab emtansine (Kadcyla)	Genentech	Humanized IgG1	HER2	Breast Cancer	ADC, Signal Inhibition, ADCC^14^	2013

**Table 2:** mAbs currently in late stage clinical trials

Name	Sponsor	Class	Target	Indication	Major mechanism	Current status
Elotuzumab	BMS/Abbott	Humanized IgG1	CS1	Multiple Myeloma	ADCC^12^^,^^16^	Phase II/III
Farletuzumab	Morphotek	Humanized IgG1	Folate Receptor α	Ovarian Cancer		Phase III
Inotuzumab ozogamicin	Pfizer/UCB	Humanized IgG4	CD22	Acute Lymphocytic Leukemia/Non-Hodgkin’s Lymphoma	ADC	Phase III
Moxetumomab pasudotox	AstraZeneca	Murine Fv	CD22	Hairy Cell Leukemia	Immunotoxin	Phase III
Naptumomab estafenatox	Active Biotech	Murine Fab	5T4	Renal Cell Carcinoma	Immunoconjugate	
Necitumumab	ImClone Systems	Human IgG1	EGFR	Non-Small Cell Lung Cancer		Phase III
Nivolumab	BMS	Human IgG4	PD1	Non-Small Cell Lung Cancer/ Renal Cell Carcinoma/Melanoma	Signal Inhibition	Phase III
Onartuzumab	Genentech	Humanized IgG1	c-Met	Non-Small Cell Lung Cancer/ Gastric Cancer	Signal Inhibition	
Racotumomab	CIMAB	Murine	GM3	Non-Small Cell Lung Cancer	Active Immunization (Vaccine)	Phase III
Rilotumumab	Amgen	Human IgG2	HGF/SF	Gastric/Gastresophageal Junction Adenocarinoma	Signal Inhibition	Phase III

Incorporation of mAbs into chemotherapeutic regimens has led to significant improvements in patient outcomes for a variety of cancers, most notably being the addition of rituximab to the standard CHOP (cyclophosphamide, doxorubicin, vincristine, and prednisone) regimen for the treatment of large B-cell lymphoma^17^^,^^18^.

In this review, we describe key considerations specific to the clinical application of mAb-based therapeutics in oncology, including pharmacologic mechanisms of action, clinical targets, and pharmacokinetic intricacies. Additionally, we summarize the clinical applications of marketed mAbs and those in late stage clinical trials.

## Pharmacokinetic (PK) considerations

All of the approved mAbs are members of the immune gamma globulin (IgG) family. When studied in healthy, human subjects, endogenous (or pooled) IgG antibodies are often found to demonstrate predictable, linear pharmacokinetics, with small volumes of distribution (~3-9 L), low rates of clearance (8-12 mL/h), and long biological half-lives (~20-25 d)^19^. However, therapeutic IgG mAbs often exhibit complex, non-linear pharmacokinetics, with substantial between- and within-patient variability. Main determinants of mAb disposition are discussed below; a more detailed description of general mAb PK/PD expectations may be found in the 2008 review by Wang *et al*.^20^.

### Target mediated drug disposition (TMDD)

For most drug molecules, the interaction between the drug and its pharmacological receptor does not contribute substantially to the kinetics of drug distribution or elimination. However, as proposed by Gerhard Levy in 1994^21^, and as described by the mathematical modeling of Mager and Jusko in 2001^22^, in cases where target-drug binding affinity is very high, the interaction between target and drug may play a significant role in drug pharmacokinetics. This phenomenon, known as target-mediated drug disposition (TMDD), leads to non-linear, saturable distribution and elimination kinetics. High affinity mAb-target binding contributes to the apparent volume of mAb distribution, as a high degree of binding leads to a high ratio of the quantity of mAb bound to cellular target proteins, relative to the concentration of mAb in blood. Additionally, in many cases, mAb-target binding precipitates the endocytosis of the mAb-target complex, with subsequent intracellular catabolism and elimination of the antibody. As such, target binding may lead to efficient mAb elimination. With increasing doses of mAb, the target becomes increasingly saturated with antibody, and this saturation leads to decreases in the apparent volume of mAb distribution and to decreases in the rate of antibody clearance (i.e., non-linear, dose-dependent pharmacokinetics).

For mAb exhibiting TMDD, intra- and inter-patient variability in target expression often is a prime determinant of pharmacokinetic variability. For example, patients with large tumor loads, and large amounts of tumor-associated target, may show much more rapid and extensive mAb distribution and elimination than observed in healthy individuals or in patients with low tumor volume. In many cases, administration of mAb leads to the destruction of cells that express the target and, consequently, mAb pharmacokinetics may be influenced by the therapeutic effects of the mAb. For example, in the clinical investigation of the pharmacokinetics of an anti-CD3 mAb, Meijer *et al*. observed that mAb elimination was more rapid for the first dose relative to the rate of mAb elimination observed for later doses (i.e., following the second, fourth, or tenth dose in a multiple-dose regimen). This finding was explained by the effect of the mAb on CD3-positive cells (i.e., depletion), which led to a reduction in target-mediated mAb clearance with increased treatment^23^. As such, for mAbs that exhibit TMDD, the ‘baseline’ target expression level, as well as the influence of mAb dosing on target expression, should be considered when evaluating mAb pharmacokinetics. Knowledge of changes in target expression due to disease progression or response to treatment may be crucial for the accurate prediction of the PK/PD of subsequent doses of mAbs.

### Tumor distribution

Due to the large molecular weight and high polarity of antibodies, mAb demonstrate very slow rates of diffusion across cell membranes and, thus, comparatively slow rates of extravasation and tissue distribution (i.e., relative to small-molecule drugs). In comparison to the distribution of mAb in healthy tissues, distribution of mAb within tumors may be further impeded due to irregularities in the tumor vasculature, and due to high interstitial pressure in tumors, as described by Jain^24^. Moreover, the high affinity binding of mAb to target proteins within solid tumors may act as a barrier to distribution, as explained by the ‘binding site barrier’ hypothesis. The impact of mAb binding on tumor distribution has been well illustrated by Fujimori *et al*., who utilized a modeling analysis demonstrating that high affinity (K_A_>1.0×10^9^ M^–1^) mAbs exhibit heterogeneous tumor distribution, with the majority of the molecules being ‘stuck’ at sites proximal to the point of extravasation within the tumor. The results of their simulations suggested that moderate affinity mAb (K_A_=5×10^7^–1×10^8^ M^–1^) would allow optimal distribution^25^. Their predictions have been supported by experimental work performed by several investigators, including Juweid *et al*., who demonstrated that, following low doses, mAb intra-tumoral distribution was limited to areas adjacent to blood vessels, and the extent of tumor distribution was enhanced following high doses of mAb, consistent with the saturation of the binding site barrier^26^.

## Pharmacologic mechanisms of action

**Figure 1:**
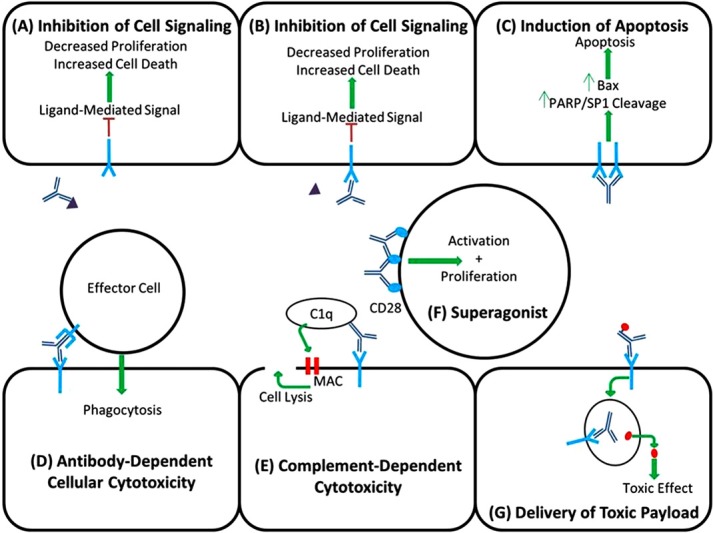
Pharmacologic Mechanisms of Action for mAbs. Panel A, Inhibition of Cell Signaling via Binding to Soluble Targe; Panel B, Inhibition of Cell Signaling via Binding to Membrane-Bound Receptor; Panel C, Direct Induction of Apoptosis; Panel D, Antibody-Dependent Cellular Cytotoxicity; Panel E, Complement-Dependent Cytotoxicity; Panel F, CD28 Superagonist; Panel G, Delivery of Toxic Payload (Antibody-Drug Conjugate, Immunotoxin, Radioimmunoconjugate).

As shown in **Table 1** and **Table 2**, mAbs have been developed to engage a wide variety of cell surface and soluble target proteins. While several factors play a role in the pharmacologic mechanism of action for mAbs, the nature of the target and its role in tumor growth are crucial players in determining how mAb will exert therapeutic effects. Therapeutic responses to mAbs may be mediated through either the Fab or Fc region of the antibody. Key pharmacodynamic mechanisms for mAbs in oncology include: inhibition of cell signaling, induction of apoptosis, antibody-dependent cellular cytotoxicity (ADCC), complement-dependent cytotoxicity (CDC), and targeting a toxic payload to tumor cells (**Figure 1**). Additionally, there has been some interest in the development of mAbs known as ‘superagonists’ that stimulate immune function to accelerate immune clearance of tumor cells. It is important to note that a single mAb may act through a combination of mechanisms to achieve anti-tumor effects.

### Inhibition of cell signaling

Monoclonal antibodies may antagonize cell signaling pathways by several mechanisms, including neutralization of soluble signaling factors (e.g., vascular endothelial growth factor, hepatocyte growth factor), binding to and blocking cell surface receptors (i.e., preventing receptor engagement with signaling factors), and by decreasing the expression of cell surface receptors. Of these mechanisms, perhaps the most interesting is the action of mAb to decrease receptor expression, which may be accomplished by ‘stripping’ the receptor from the cell surface or by accelerating the internalization and catabolism of the receptor. Dose requirements for the inhibition of signaling may be, in some cases, estimated based on the rate of production of the antibody target, whether it is a receptor or a soluble ligand. In most cases, blockade of cell signaling will not require engagement of the Fc domain of the mAb, and may be accomplished with administration of Fab fragments. For example, early work investigating the use of anti-EGFR mAbs in the treatment of cancer demonstrated that (Fab)_2_ fragments were able to produce a dose-dependent reduction in tumor growth in a xenograft model, supporting the hypothesis that the activity of this mAb did not require Fc-dependent effector mechanisms^27^.

### Antibody-dependent cellular cytotoxicity (ADCC)

Early studies performed using cultured human monocytes in the presence and absence of murine IgG2a demonstrated that the ability of monocytes to kill tumor cells was significantly increased in the presence of IgG^28^. This phenomenon, which has been dubbed Antibody-Dependent Cellular Cytotoxicity (ADCC), is mediated by the interaction between the Fc region of an antibody and FcγRIIIa receptors present on the surface of immune cells. Briefly, mAb may bind a cell surface target via its Fab region, and then engage leukocytes expressing FcγRIIIa via the Fc region of the mAb, leading to subsequent cell killing.

One example demonstrating the importance of the FcγR-mAb interaction has been provided by Cartron *et al*.^29^, who investigated the efficacy of rituximab in a panel of lymphoma patients. This team demonstrated that patients expressing a low affinity variant of FcγRIIIa, which contains a phenylalanine residue at position 158, received less benefit (i.e., a shorter survival time) from rituximab treatment than patients who possess a high affinity variant of the receptor, with valine at position 158. This work strongly suggests that a significant fraction of the benefit provided by rituximab is resultant from ADCC.

### Complement-dependent cytotoxicity (CDC)

The ability of immunoglobulin molecules to recruit complement to mediate cell killing has been appreciated for several decades. Briefly, following mAb binding to a cell-surface target, Fc domains of the mAb may bind to soluble C1q (i.e., complement fixation), leading to stimulation of the complement pathway, and ultimately cell death. Complement fixation requires relatively high densities of mAb on the cell surface, as it has been estimated that C1q fixation requires separation of Fc domains by no more than ~40 nm^30^. In one example of the significance of CDC for mAb treatment of cancer, Capone and colleagues generated two monoclonal antibodies against breast cancer targets, and investigated possible mechanisms to explain their *in vivo* tumor cell killing. *In vitro* cell killing, for each mAb, required complement, implicating CDC as the primary mechanism of cell killing^31^. *In vitro* studies performed with matched chimeric mAbs of various subclasses have demonstrated that the IgG1 subclass has the greatest ability to induce cell death via CDC^32^.

### Induction of apoptosis

Monoclonal antibody binding to cell surface receptors may lead to the induction of cell death via apoptotic pathways. For example, work by Trauth *et al*.^33^ showed that anti-APO-1 mAbs induce apoptosis in tumor cells, *in vitro* and *in vivo*, in a manner distinct from ADCC and CDC. Briefly, they noted that cell death could occur after mAb binding under complement-free and serum-free conditions, and that the pattern of cell death was consistent with apoptosis and not necrosis, suggesting that the mAb-target interaction directly led to the induction of apoptosis.

### Superagonists

In addition to the mechanisms of action discussed above, there has been an interest in developing immunomodulatory mAbs, which function as “superagonists”. Briefly, T cell stimulation typically requires a signal from the T Cell Receptor (TCR) and a co-stimulatory signal from CD28^34^. However, superagonist antibodies, such as the anti-CD28 mAb TGN-1412, have demonstrated an ability to stimulate T cell proliferation without TCR engagement^35^, thereby creating a possible mechanism for mAbs to increase immune-mediated clearance of cancers. However, superagonist mAbs may bring significant risks, as evidenced by the first-in-man investigation of TGN-1412. Briefly, the anti-CD28 mAb was dosed to six healthy volunteers, who all experienced a cytokine storm shortly after administration of the mAb, leading to severe side effects in all of the subjects^36^. It is possible that revised dosing schemes, possibly targeted low levels of mAb binding to stimulatory receptors, may allow for the desired anti-cancer effect without risk for toxicities associated with hyper-stimulation.

### Delivery of toxic payloads (immunoconjugates)

The use of mAbs to deliver a highly potent payload to tumor sites is perhaps the epitome of Ehrlich’s ‘magic bullet’ hypothesis. There are three major types of constructs which fall into this broad category: antibody-drug conjugates (ADCs), immunotoxins, and radioimmunoconjugates. Briefly, each construct is designed to employ the target specificity of a mAb to deliver a toxic payload selectively to tumor cells, potentially delivering high potency and low toxicity. Due to the complexity of these delivery systems, there are many potential issues which must be considered when developing an immunoconjugate, which have been outlined in detail in other reviews^37^^,^^38^.

The selective delivery of a small molecule chemotherapeutic agent to tumors using mAbs has been tested preclinically and clinically for several decades. The earliest examples of ADCs used mAbs to target clinically approved chemotherapeutics to tumors. However, these constructs often failed in clinical trials due to insufficient potency of the payload. A notable example of this failure was BR96-DOX, which showed remarkable antitumor activity in preclinical models^39^, but had unacceptable efficacy and toxicity profiles in clinical trials^40^.

Currently, ADC payloads are often selected from chemotherapeutic agents that have demonstrated unacceptable pharmacokinetic or toxicity profiles for clinical development, thereby ‘resurrecting’ drug molecules that have been previously discarded. The most commonly clinically utilized agents at this time are maytansinoids, calicheamicins, and monomethyl auristatin E (MMAE).

While ADCs utilize small molecule chemotherapeutics as the cytotoxic agent, immunotoxins use highly potent bacterial and plant toxins to exert their antitumor effect. Currently, there are two anti-CD22 immunotoxins in clinical development^41^. Other payloads which have been investigated in the literature include ricin-like toxins^42^, as these molecules are extremely potent and are believed to have the capacity to induce apoptosis with only a single molecule entering the cytosol.

Radioimmunoconjugates, the final class of immunoconjugates, employ mAbs as targeting agents for selective delivery radionuclides to tumor cells. Several radionuclides have been investigated in preclinical and clinical trials; the two clinically approved agents employ ^90^Y (β emitter) and ^131^I (γ emitter). In many therapeutic protocols utilizing radioimmunotherapy, patients are pre-dosed with unlabeled (i.e., ‘cold’) mAb, followed by the administration of the radioimmunoconjugate. This strategy often allows minimization of off-target toxicities, via saturation and/or depletion of the target protein on healthy cells that may be associated with low density target expression. As such, pre-dosing often allows more selective uptake of radioimmunoconjugate in tumor cells, and improved pharmacokinetics of the conjugate^43^.

## Currently marketed mAbs in oncology

FDA-approved mAbs used in oncology are summarized in **Table 1**. Important PK/PD considerations for each mAb have been detailed below.

### Alemtuzumab

Alemtuzumab (Campath) is an anti-CD52 mAb approved for use in B-cell chronic lymphocytic leukemia (B-CLL). The PK of alemtuzumab has been described as being non-linear, with the maximal rate of elimination demonstrating co-variation with white blood cell counts, consistent with TMDD (note that CD52 is expressed on leukocytes)^44^. In addition to non-linear PK, alemtuzumab may display time- or treatment-dependent kinetics, where half-life increases after initial elimination of target-expressing cells. Key mechanisms of action of alemtuzumab include induction of apoptosis and CDC^6^.

In Phase III clinical trials, alemtuzumab was compared to chlorambucil, in previously untreated B-CLL patients. Those subjects who received alemtuzumab showed a 42% reduction in the risk of progression-free survival, as well as a significant (*P*<0.0001) improvement in overall response rates^45^. Additionally, other trials have demonstrated that this mAb is effective in treating patients with p53 mutations and deletions, which render chlorambucil treatment ineffective^46^. Alemtuzumab was withdrawn from the market in 2012, but its sponsors are seeking to rebrand it as a treatment for multiple sclerosis.

### Bevacizumab

Bevacizumab (Avastin) is an anti-VEGF mAb that is approved for use in several cancers, including metastatic colorectal cancer (mCRC), non-squamous, non-small cell lung cancer (NSCLC), glioblastoma, and metastatic renal cell carcinoma. This mAb functions by binding to circulating VEGF, blocking its ability to bind to its target receptor, and blocking VEGF-mediated stimulation of pro-angiogenic signaling pathways. As is typical for mAbs which bind soluble ligands, bevacizumab displays linear PK, with a terminal half-life of ~20 days^47^. In trials, this mAb was added on to a standard chemotherapy combination of irinotecan, bolus fluorouracil, and leucovorin (IFL) to determine its efficacy in mCRC. Patients which were on the bevacizumab arm of the treatment showed improvements in all clinical efficacy endpoints tested in the study^48^.

### Brentuximab vedotin

Brentuximab vedotin (Adcetris) is an anti-CD30 ADC approved as a second or third line therapy for the treatment of Hodgkin’s lymphoma (HL) and systemic anaplastic large cell lymphoma (ALCL). This construct consists of an anti-CD30 mAb linked to MMAE, which is a highly potent anti-mitotic agent. The clinical PK of brentuximab vedotin has been reported as ‘approximately proportional to dose’, with a half-life of 4-6 days for the intact ADC and 3-4 days for MMAE^49^. Due to overwhelmingly positive results in two Phase II clinical trials^50^^,^^51^, this ADC was granted an accelerated approval. Results from these studies demonstrated a 75% response rate in HL^51^ and an 86% response rate in ALCL^50^, indicating that this drug has great potential in the treatment of these cancers.

### Cetuximab

Cetuximab (Erbitux) is an anti-EGFR mAb which is approved for the treatment of squamous cell carcinoma of the head and neck (SCCHN) and *K-Ras* mutation negative, EGFR positive, mCRC. In a dose-ranging (50-500 mg/m^2^) study, cetuximab clearance was found to range from 20.0-83.7 mL/h/m^2^, indicating the presence of a saturable elimination pathway for this mAb, likely consistent with TMDD^52^. Cetuximab has been reported to exert its anti-tumor properties via signal inhibition, ADCC, and CDC^8^. When added to standard radiotherapy in patients diagnosed with SCCHN, cetuximab increased overall survival by 19.7 months and progression-free survival by 9.5 months, indicating benefit compared to the standard of care^53^. *K-Ras* status has been investigated as a predictor of response to cetuximab, and trials have shown that patients positive for mutations in *K-Ras* have significantly lower responses when treated with cetuximab, likely due to the constitutive activation status of the variant protein^54^.

### Denosumab

Denosumab (Xgeva) is an anti-RANKL mAb approved for the treatment of bone metastases from solid tumors and for unresectable giant cell bone tumors. Binding of denosumab to RANKL prevents interaction with RANK, thereby preventing osteoclasts from resorbing bone. The pharmacokinetics of denosumab have been reported as non-linear, with a maximal clearance value of 85 mL/h, and with saturation of the target-mediated pathway being achieved with doses of 120 mg/month^55^.

A study in patients with breast cancer bone metastases demonstrated that denosumab was superior to the bisphosphonate zoledronic acid in the prevention of skeletal-related events such as pathological fractures, spinal cord compression, and bone surgery/radiation^56^. This indicates that use of this mAb may help to reduce some of the consequences of bone metastases in patients, improving their quality of life.

### Ibritumomab tiuxetan

Ibritumomab tiuxetan (Zevalin) is an anti-CD20 radioimmunoconjugate indicated in the treatment of non-Hodgkin’s lymphoma (NHL). Administration of this drug is performed by first infusing rituximab followed by ibritumomab tiuxetan conjugated with either ^111^In (imaging) or ^90^Y (treatment). Clinical trials showed an increase in progression-free survival of 1.1 months and an increased complete response rate when treating patients with ^90^Y-ibritumomab tiuxetan compared to rituximab treatment, which indicates that delivery of the radioisotope allows for improved outcomes compared to a ‘naked’ mAb delivered to the same target^57^.

### Ipilimumab

Ipilimumab (Yervoy) is an anti-CTLA-4 mAb indicated for the treatment of unresectable or metastatic melanoma. In metastatic melanoma patients, ipilimumab pharmacokinetics were found to be linear over a dose range of 3-10 mg/kg, with an average clearance value of 14.9 mL/h^58^. Because this mAb targets an antigen expressed on T-cells, distributional challenges are not likely to be a significant determinant of its efficacy. Binding of ipilimumab to CTLA-4 relieves inhibitory signals on T-cell proliferation, thereby improving immune function in patients. Effectively, ipilimumab treatment serves to counteract the immune evasion mechanisms utilized by tumors to ensure their continued survival.

Patients with unresectable stage III or IV melanoma were treated with ipilimumab and/or a gp100 peptide vaccine, and it was observed that ipilimumab alone improved overall survival by 3.6 months compared to vaccine alone (6.4-10.0 months)^59^. Additionally, early clinical trial results indicated that treatment with ipilimumab led to an increase in lymphocyte activation markers, indicating improved immune functions in patients receiving mAb therapy^60^.

### Ofatumumab

Ofatumumab (Arzerra) is an anti-CD20 mAb currently approved for use in treatment of chronic lymphocytic leukemia (CLL). In patients, ofatumumab displays both dose- and treatment-dependent pharmacokinetics over a dose range of 500-2,000 mg. On the first dose, clearance ranged from 65-215 mL/h, while after the fourth dose, clearance decreased to 10-28 mL/h^61^. Briefly, this suggests that the elimination of ofatumumab is target-mediated, and that wipeout of CD20-positive cells after early doses contributes to a slower clearance on subsequent doses. It has been suggested that the primary mechanisms by which ofatumumab kills cancer cells are ADCC and CDC^11^. In trials as a single agent in CLL patients refractory to standard treatments (fludarabine), ofatumumab improved response rates from 23% to 47%-58% along with a median progression-free survival time of six months^62^. Additionally, trial results in follicular lymphoma have indicated that ofatumumab has some activity in rituximab-refractory patients^63^.

### Panitumumab

Panitumumab (Vectibix) is an anti-EGFR mAb indicated for use in the treatment of mCRC. Clearance of panitumumab is markedly non-linear, approaching values of ~75 mL/d/kg at low doses (0.75 mg/kg), and decreases to ~4 mL/d/kg at higher doses (>2 mg/kg)^64^. Interestingly, panitumumab is eliminated more slowly than cetuximab, indicating that the target-mediated pathway may be less relevant for this mAb^64^. Additionally, the binding site barrier has been reported as relevant in preclinical models, with deeper penetration occurring into tumors at higher doses (500 µg) and later time points (96 h)^65^. The reported mechanisms of action for panitumumab include signal inhibition, ADCC, and CDC^10^.

Clinical trials in mCRC patients who had progressed after standard therapy, compared to best supportive care alone, indicated that panitumumab improved median progression-free survival from 7.3 to 8.0 weeks^66^. Additionally, as with other anti-EGFR mAbs, wild type *K-Ras* is necessary for response to treatment^67^.

### Pertuzumab

Pertuzumab (Perjeta) is an anti-HER2 mAb indicated for the treatment of metastatic breast cancer. The PK of pertuzumab was found to be linear in the dose range of 0.5-15 mg/kg (mean clearance has been reported to be 0.214 L/d)^68^. The interaction of pertuzumab with HER2 is such that it blocks the interaction of HER2 and HER3, preventing dimerization and subsequent intracellular signaling^13^. In addition to this direct, Fab-mediated mechanism, pertuzumab also may induce cell death via ADCC^13^. Because pertuzumab targets a different motif in HER2 than trastuzumab, combination therapy was investigated to determine if there could be synergistic benefits. Addition of pertuzumab to trastuzumab and docetaxel therapy led to an increase in progression-free survival by 6.1 months (12.4-18.5 months), producing a clear clinical benefit when added to standard therapy^69^.

### Rituximab

Rituximab (Rituxan) is an anti-CD20 mAb indicated as a therapy for treatment of NHL and CLL and was the first mAb approved by the FDA for use in oncology. In clinical trials for NHL, rituximab was found to have non-stationary pharmacokinetics, with clearance decreasing from 38.2 mL/h after the first dose to 9.2 mL/h after the fourth dose^70^. This observation may be due to a reduction in TMDD caused by wipeout of CD20-positive cells after the initial infusion. In clinical trials, addition of rituximab to the standard CHOP-21 chemotherapy regimen was associated with an improvement in 3-year progression-free survival (85% *vs*. 68%)^17^. In a different study, lymphoma patients treated with rituximab alone had an overall response rate of 50%, with a median duration of response of 8.6 months^71^.

### Tositumomab

Tositumomab (Bexxar) is an anti-CD20 mAb indicated for the treatment of relapsed or refractory NHL, and is administered first as a ‘cold’ mAb, followed by administration of a ‘hot’ ^131^I-labeled mAb. It was noted in clinical trials that patients with a greater tumor burden were associated with increased volume of distribution, faster clearance, and shorter half-life of tositumomab^72^, indicating that TMDD is likely relevant in the pharmacokinetics of this drug. As this radioimmunotherapy regimen is not intended for first-line treatment of NHL, the pivotal clinical trial evaluated tositumomab compared to standard last qualifying chemotherapy regimens. In this trial, patients receiving tositumomab had a median duration of response of 6.4 months, compared to 3.4 months in the control group, with 3% of patients achieving a complete response^73^. As of February 2014, tositumomab will be withdrawn from the market in the U.S. and Canada, due to a manufacturer’s decision^74^.

### Trastuzumab

Trastuzumab (Herceptin) is an anti-HER2 mAb approved for the treatment of breast cancer, metastatic gastric cancer, and metastatic gastroesophageal junction adenocarcinoma. The half-life of trastuzumab has been observed to range from 1.1 days (10 mg dose) to 23 days (500 mg dose) in clinical trials^75^. Additionally, population pharmacokinetic modeling has suggested that clearance of trastuzumab is directly related to shed extracellular domain of HER2 and has a weaker association with the number of tumor metastases^75^. In mice, tumor distribution was found to be more uniform at higher doses and at later time points, suggesting that saturation of the binding site barrier may be crucial in optimizing the efficacy of trastuzumab^76^.

Phase III clinical trials investigated the potential benefits of adding trastuzumab to standard chemotherapy in previously untreated breast cancer patients with HER2-overexpressing tumors. The trial results indicated that addition of trastuzumab was associated with a 4.8-month increase in overall survival (20.3-25.1 months) and a 2.8-month increase in progression-free survival (4.6-7.4 months)^77^.

### Trastuzumab emtansine

Trastuzumab emtansine (Kadcyla) is an anti-HER2 ADC indicated for treatment of metastatic breast cancer. This ADC consists of the anti-HER2 mAb trastuzumab linked to mertansine, a maytansinoid which exerts its cytotoxic effect via tubulin binding. In addition to delivery of mertansine, this ADC retains the mechanisms of action associated with the ‘naked’ mAb, trastuzumab (signal inhibition and ADCC)^14^. In dose-escalation studies, there was an observed trend towards faster clearance at doses less than 1.2 mg/kg/3 weeks (CL=21.1-27.8 mL/d/kg); however, linear PK was observed at higher doses (CL=7.13-12.7 mL/d/kg), indicating a saturable clearance pathway78. Additionally, the observed free DM-1 (payload) concentrations did not exceed 25 ng/mL, indicating that the conjugate is stable in plasma^78^.

In trials with patients diagnosed with advanced breast cancer, trastuzumab emtansine increased progression free survival relative to standard of care (lapatinib plus capecitabine) by 3.2 months (6.4-9.6 months) and median overall survival by 5.8 months (25.1-30.9 months)^79^. Additionally, the ADC was shown to have efficacy in patients whose disease had progressed after prior HER2-targeted therapy (progression-free survival =4.6 months)^80^.

## mAbs in late-stage clinical trials

In addition to the currently marketed mAb products, there is a rich pipeline of products that are currently being investigated in clinical trials. Here we summarize mAb-based products that are in late-stage (Phase II and Phase III) clinical trials for cancer indications to give an overview of products that may be clinically available in the next few years.

### Elotuzumab

Currently in Phase III clinical trials, elotuzumab is an anti-CS1 (CD2 subset 1) mAb being investigated as a treatment option for multiple myeloma. Results of a dose-escalation study indicate that elotuzumab displays clear non-linear pharmacokinetics, with clearance decreasing from 71.4 to 15.7 mL/h over the dose range of 0.5-20 mg/kg^81^. Phase I clinical trial results indicated that this mAb has efficacy in treatment of multiple myeloma in combination with lenalidomide and dexamethasone (objective response rate of 82%)^82^.

### Farletuzumab

Farletuzumab is an anti-folate receptor α (FRA) mAb that is being investigated for use in ovarian cancer, along with other epithelial cancers. Early clinical trial results in relapsed platinum sensitive ovarian cancer indicated that as a single agent farletuzumab induced stable disease at best in 30% of patients, while in combination with carboplatin and taxane 95% of patients achieved stable disease or better^83^. However, when this drug progressed into Phase III clinical trials, patients did not show a statistically significant improvement in progression-free survival relative to the control arm^84^, leaving the future of farletuzumab in ovarian cancer treatment uncertain.

### Inotuzumab ozogamicin

Inotuzumab ozogamicin is an ADC directed against CD22 which has progressed into Phase III clinical trials for the treatment of NHL and acute lymphocytic leukemia (ALL). In this construct, ozogamicin (a calicheamicin derivative) is the toxic payload used to destroy tumor cells. Phase I clinical trial data indicates that this ADC displays non-stationary PK, with decreased clearance after multiple dosing, relative to the first dose, indicative of the modulation of a target-mediated pathway with initial doses^85^^,^^86^.

Trials for the ADC in the treatment of NHL have been halted as of May 2013, as the drug in combination with rituximab was not likely to result in a significant improvement in overall survival, based on the planned interim analysis^87^. However, trials for other conditions, such as ALL, are ongoing and have shown promising results, with 58% of patients achieving a bone marrow complete response in a published study^88^.

### Moxetumomab pasudotox

Moxetumomab pasudotox is an anti-CD22 immunotoxin consisting of an Fv as the targeting moiety fused to Pseudomonas exotoxin-A, which is being investigated in Phase III clinical trials for hairy cell leukemia (HCL). Published Phase I trial data is promising with an overall response rate of 86%^89^. Pharmacokinetic analysis of data has suggested that tumor burden is a significant covariate on clearance, demonstrating that TMDD may be important in the *in vivo* behavior of this drug^90^.

### Naptumomab estafenatox

Naptumomab estafenatox is an anti-5T4 fusion protein consisting of a Fab fragment fused to staphylococcal enterotoxin E, which is being studied for use in renal cell carcinoma. This construct is proposed to function as a superantigen, recruiting immune effectors to the target site^91^. Within individual cycles of therapy, it was noted that the PK of naptumomab estafenatox was linear^92^. When comparing the first and second cycles of therapy, clearance was dramatically increased after the second dose of the drug (increased from 0.11 to 6.39 L/h/kg), which the investigators suggest was due to formation of antibodies against the construct^92^. Phase I trials support this mechanism as post-treatment tumor biopsies had significant T cell infiltration, and the construct has measurable anti-tumor activity in the clinic^92^.

### Necitumumab

Necitumumab is an anti-EGFR mAb being developed for use in the treatment of NSCLC. In clinical trials, necitumumab displayed both dose- and treatment-dependent PK, with clearance values after the first dose (100-1,000 mg/week) ranging 13.9-53.2 mL/h, whereas after the final dose, clearance ranged from 1.45-40.2 mL/h^93^. Eli Lilly has recently announced that Phase III trials in stage IV NSCLC, where necitumumab was added to standard chemotherapy, have met the primary endpoint of increased overall survival, and they intend to submit data for regulatory approval in 2014^94^.

### Nivolumab

Nivolumab is an anti-programmed cell death receptor 1 (PD1) mAb which is currently in clinical trials for the treatment of NSCLC, renal cell carcinoma, and melanoma. The PK of nivolumab shows modest non-linearity, with a terminal half-life of 12 days at dose levels less than 3 mg/kg and a half-life of 20 days at a dose of 10 mg/kg, indicating that there is a saturable clearance pathway for this mAb^95^. To date, the most striking results have been observed when nivolumab was administered in combination with ipilimumab in stage III or IV melanoma patients. Clinical activity was observed in 65% of patients receiving the combination, and 53% of patients who received the maximum dose had a tumor reduction of greater than 80%^96^.

### Onartuzumab

Onartuzumab is an anti-hepatocyte growth factor receptor (c-Met) monovalent mAb in trials for use in NSCLC and gastric cancer. At dose levels greater than 4 mg/kg, the PK appears to be linear; however, at a low dose (1 mg/kg), clearance is approximately two-fold greater than at the higher doses, indicating that there may be a readily saturated TMDD pathway^97^. However, population PK modeling based on Phase I and II clinical trials indicates that a 15 mg/kg dose every three weeks is adequate for the desired exposure, thereby minimizing the influence of the target-mediated clearance pathway^97^.

### Racotumomab

Racotumomab is an anti-GM3 mAb in Phase III clinical trials as a cancer vaccine for advanced NSCLC. Phase I clinical trial data in patients with NSCLC showed that treatment with racotumomab (4+ doses) produced a specific antibody response against both the mAb and against the specific target, along with generating a favorable survival profile^98^.

### Rilotumumab

Rilotumumab is an anti-hepatocyte growth factor (HGF) mAb being investigated for the treatment of gastric and gastroesophageal cancers. Results from dose escalation studies (0.5-20 mg/kg) indicate that rilotumumab displays linear PK in man, with the average clearance being 0.141 mL/h/kg and no clear dose-dependent changes observed^99^.

## Conclusion

In this review, we have summarized the pharmacokinetics and pharmacodynamics of monoclonal antibodies used for oncologic indications, including mechanisms of action. Monoclonal antibodies may be considered to be the most important class of anti-cancer agents, with 14 mAbs in current clinical use, and with many more in development. This drug class, which achieves effects through a variety of mechanisms, provides several benefits over traditional small-molecule chemotherapeutic agents, including slow rates of elimination (thus allowing infrequent dosing), high efficacy, and low off-target toxicity. Based on the promise of agents in development, it is anticipated that anti-cancer mAbs will continue to grow in importance over the next 5-10 years.
